# Development of a lipoprotein(a)-based model for predicting progression-free survival and grade3/4 adverse events in driver gene negative metastatic lung adenocarcinoma patients with PD-L1 TPS <50%

**DOI:** 10.3389/fimmu.2026.1808231

**Published:** 2026-04-24

**Authors:** Sicong Li, Yiyuan Cui, Yijing Yan, Hao Li, Yue Jin, Yufan Chen, Jingjie Yu, Li Feng

**Affiliations:** 1 National Cancer Center/National Clinical Research Center for Cancer/Cancer Hospital, Chinese Academy of Medical Sciences and Peking Union Medical College, Beijing, China; 2 School of Pharmacy, Peking University Health Science Centre, Beijing, China; 3 Beijing University of Chinese Medicine, Beijing, China

**Keywords:** chemo-immunotherapy, grade 3/4 adverse events, lipoprotein(a), lung adenocarcinoma, progression-free survival

## Abstract

**Objective:**

This study evaluated the value of lipoprotein(a) (LPA) in lung adenocarcinoma (LUAD) patients receiving first-line chemoimmunotherapy and developed a model to predict progression-free survival (PFS) and grade 3/4 adverse events (G3/4 AEs).

**Methods:**

A prospective cohort study was conducted on driver gene negative metastatic LUAD patients with PD-L1 TPS <50%, who received first-line chemoimmunotherapy. The data were randomly sampled into training and internal validation sets following a 7:3 proportion. We constructed a prognostic model for progression-free survival (PFS) via LASSO and multivariate Cox regression analyses. We explored five methods—random forest, AdaBoost, elastic-net, LASSO, and support vector machine (SVM)—to develop a prediction model for G3/4 AEs.

**Results:**

A total of 227 patients completed the follow-up. The AUC was 0.78(0.62-0.94) for 365-day PFS in the training cohort and 0.95(0.84-1.00) in the internal validation cohort. The serum LPA level independently predicted disease progression in patients receiving first-line chemoimmunotherapy. AdaBoost outperformed other machine learning methods in terms of accuracy, precision, recall, and F1 scores on both the training and validation sets, leading to its selection for the final G3/4 AE prediction model.

**Conclusion:**

High LPA expression in the serum was a risk factor for metastatic driver gene-negative lung adenocarcinoma patients receiving first-line chemoimmunotherapy. Our models had favorable value in predicting PFS and G3/4 AEs, which might assist in identifying patients less likely to benefit from initial chemoimmunotherapy.

## 1 Introduction

Lipoprotein(a), also known as LPA, is a type of cholesterol particle that closely resembles low-density lipoprotein (LDL) cholesterol and is attached to a protein known as apolipoprotein(a) (1). Serum LPA levels are significantly linked to the risk of atherosclerosis, thrombosis, and stroke (2). A cross-sectional study reveals significantly elevated plasma LPA levels in male primary lung cancer patients compared with healthy male controls (P = 0.004) (3). Although significant positive linear correlation is found between peripheral blood lipoprotein(a) levels and baseline tumor diameter in non-small cell lung cancer (NSCLC) patients (P = 0.002) (4), the relationship between lipoprotein(a) expression levels and prognosis in lung adenocarcinoma patients receiving various treatments is not well understood. Compared with chemotherapy, programmed cell death protein-1 (PD-1) inhibitors have notably improved clinical outcomes in patients with metastatic driver-gene-negative NSCLC. Beyond its established role in cardiovascular disease, LPA carries oxidation-specific epitopes (OSEs) that function as danger-associated molecular patterns (DAMPs), capable of activating macrophage (5–7). In cardiovascular contexts, LPA promotes endothelial inflammation, and induces the expression of the chemokine C-C motif chemokine ligand 2 (CCL2), thereby enhancing monocyte recruitment (5, 7, 8). The functional consequences of these mechanisms in tumor biology are unclear: DAMPs can either enhance antitumor immunity (by promoting dendritic cell maturation and antigen presentation) or contribute to tissue toxicity (through excessive inflammation), but CCL2 has emerged as a critical mediator of immunotherapy resistance (6). The relationship between LPA and the efficacy or toxicity of chemoimmunotherapy has not been investigated. Chemoimmunotherapy has become the standard treatment regimen for advanced NSCLC; however, efficacy varies considerably among patients, necessitating reliable biomarkers to guide clinical decision-making. To our knowledge, predictive models are still lacking for metastatic LUAD patients who are driver-gene-negative with a PD-L1 tumor proportion score (TPS)<50% and who are receiving first-line immune checkpoint inhibitor (ICI) therapy combined with platinum-based doublet chemotherapy (9). This treatment is the current standard for metastatic NSCLC patients with PD-L1 TPS <50% and driver-gene-negative status (10–16). We concentrate on LUAD patients because their survival rates are poorer than those of patients with lung squamous cell carcinoma (LUSC). A meta-analysis of 17 trials on advanced driver-gene-negative NSCLC reveals that LUSC patients have better PFS outcomes with chemoimmunotherapy (HR 0.51, 95% CI 0.39–0.62) than nonsquamous carcinoma patients, primarily LUAD patients (17). This study prospectively examines metastatic LUAD patients undergoing first-line chemoimmunotherapy to assess the prognostic significance of serum LPA levels and develops models for 1-year PFS and G3/4 AEs.

## 2 Methods and materials

The Ethics Committee of the National Cancer Center/National Clinical Research Center for Cancer/Cancer Hospital, Chinese Academy of Medical Sciences and Peking Union Medical College approved this prospective cohort study (Approval number 24/193-4473).

### 2.1 Inclusion and exclusion criteria

**Figure 1:**
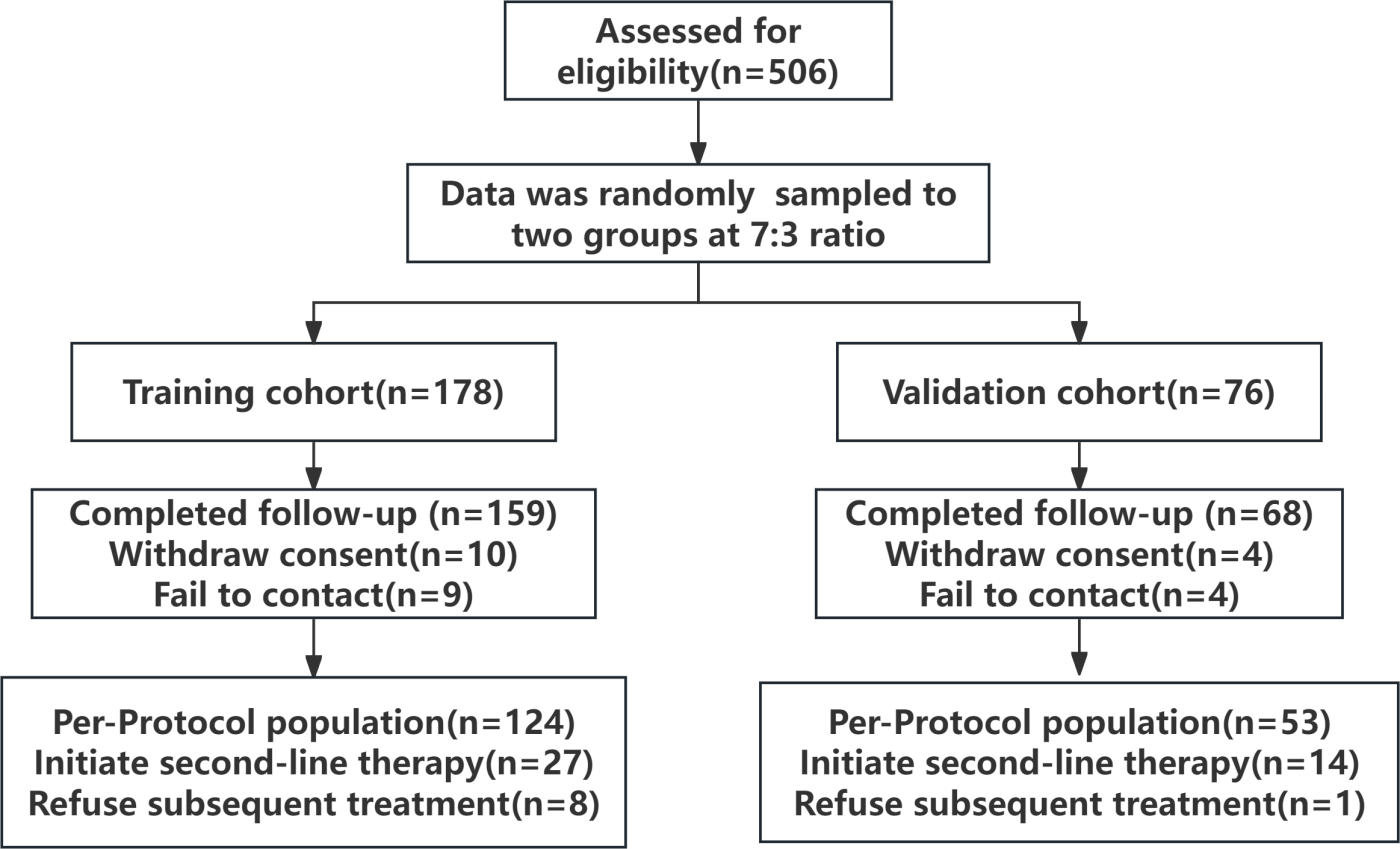
Flow chart of patient screening and grouping.

Eligible participants were those hospitalized for the first time between April 1, 2023, and April 30, 2024, meeting the following criteria: (1) histologically confirmed lung adenocarcinoma; (2) TNM stage IV; (3) at least one measurable lesion per RECIST Version 1.1; and (4) first-line treatment with a PD-1 inhibitor and pemetrexed plus carboplatin or cisplatin for 4 to 6 cycles, followed by maintenance therapy with pemetrexed and a PD-1 inhibitor for at least 2 cycles. Participants aged 18–75 years, of any sex, who voluntarily enrolled in the study and provided informed consent, had an Eastern Cooperative Oncology Group performance status (ECOG PS) of 0 or 1 and a PD-L1 TPS less than 50%. Patients were excluded if they (1) had documented actionable oncogenic driver mutations (18); (2) underwent salvage or conversion surgery postsystemic therapy; (3) had other primary solid or hematologic malignancies; (4) had incomplete baseline imaging (chest CT, abdominal ultrasound, bone scan, brain MRI, or PET-CT) or missing LPA biochemical and clinical demographic data; (5) had severe comorbidities, such as cardiovascular or cerebrovascular disease, advanced hepatic or renal impairment, or autoimmune disorders, due to safety concerns with immunotherapy or chemotherapy; (6) were pregnant or lactating; (7) had a history of organ transplantation; or (8) had immunodeficiency diseases. Among the 506 assessed patients, 254 were ultimately enrolled in the study. In a 7:3 ratio, data from participants were randomly sampled to a training cohort (n = 176) or a validation cohort (n = 78), with 159 and 68 patients completing follow-up, respectively (**Figure 1**).

### 2.2 Evaluation of therapeutic outcomes and follow-up

The patients were observed for a median duration of 11.36 months (341 days). Progression- free survival (PFS) was assessed by the duration from the index date to the earliest occurrence of (1) radiologically confirmed progressive disease (PD); (2) death from any cause, whichever occurred first. Throughout the treatment period, regular monitoring was performed, with evaluations every two to three cycles via laboratory tests and imaging studies. For patients who commenced a new treatment line, progression versus censoring was determined strictly by the radiographic findings from the last imaging assessment performed prior to treatment change. Specifically, the date of this pre-treatment radiographic evaluation served as the PFS event date (progression) or censoring time point, irrespective of the decision to initiate new therapy. Tumor response was assessed by investigators following RECIST version 1.1 guidelines. The secondary endpoints were the ORR and grade 3/grade 4 adverse events (G3/4 AEs). G3/4 AEs were defined on the basis of the Common Terminology Criteria for Adverse Events (version 5.0). Study follow-up was conducted prospectively up to June 30, 2025. Patients underwent routine clinical assessments as part of standard oncological care, including outpatient visits and telephone consultations, with data extracted from electronic medical records. The index date was defined as the date of first-line treatment initiation. PFS was calculated from the index date until the date of radiographic progression, all-cause death, whichever occurred first. For patients who did not progress, censoring occurred at the date of last imaging assessment; for progressors, the endpoint was the date of first documented radiographic progression. Patients were considered lost to follow-up if they had no outpatient visits, imaging records, or telephone contact for ≥3 months prior to June 30, 2025, or if they formally withdrew consent for data usage.

### 2.3 Sample size calculation

In the CameL study involving advanced nonsquamous NSCLC patients, 12.7% of those treated with first-line camrelizumab and chemotherapy experienced disease progression after a median follow-up of 11.9 months (19). We assumed a hazard ratio of 1.75 for the prognostic model score and a 1-year progression event rate of 12.7%. With α set at 0.05, the study power at 80%, and the confidence level at 95%, we calculated the sample size for this cohort study via the survival package (version 3.6--4) and the sample size function, which yielded a required sample size of 227 participants. The final sample size needed, accounting for a 10% dropout rate, was calculated to be 252 participants.

### 2.4 Data collection and definitions

PD-L1 tumor proportion scores were categorized on the basis of the 50% and 1% thresholds. Baseline hyperlipidemia data were systematically extracted from electronic records. Statin usage was defined as the administration of any statins for a cumulative period exceeding 120 days during the follow-up (20). Stage IV patients were defined according to the TNM staging criteria outlined by the Chinese Society of Clinical Oncology (CSCO) (21). Individuals were classified as ‘nonsmokers’ if they had consumed fewer than 100 cigarettes or equivalent amounts of tobacco in their lifetime and as ‘smokers’ if they had consumed 100 or more. Baseline serum LPA concentrations were measured via a latex-enhanced immunoturbidimetric test, with data obtained from electronic medical records. All values were recorded in mg/dL. The immunotherapy regimen was as follows: intravenous infusion of camrelizumab or tislelizumab every 3 weeks. Combination chemotherapy included pemetrexed with either carboplatin or cisplatin, which was also administered every 3 weeks. After completing 4–6 cycles of PD-1 inhibitor therapy combined with platinum-based doublet chemotherapy, patients transitioned to receive at least 2 additional cycles of pemetrexed plus PD-1 inhibitor therapy. The per-protocol (PP) population included participants who closely followed the study protocol, which meant that they received their assigned treatment as planned and completed all necessary follow-up procedures without significant violations. Baseline metastatic sites were defined as all radiographically confirmed metastatic lesions present prior to the initiation of first-line chemoimmunotherapy.

### 2.5 Construction of a prognostic nomogram for PFS

The survival (v3.6-4) and survminer (v0.4.9) were used to perform survival analyses. Initially, each potential covariate was evaluated via a univariate Cox model to determine its hazard ratio (HR) and 95% confidence interval (CI) for PFS. We subsequently used glmnet (version 4.0-2) to perform variable selection via LASSO regression with 10-fold cross-validation. Variable corresponding to lambda.min were retained, yielding three nonzero-coefficient variables. The final model was constructed via multivariate Cox regression. The resulting HRs and CIs were plotted as forest plots generated via ggplot2 package (version 3.5.1). A prognostic nomogram was subsequently built with the rms package (version 4.4.0). This graphical model translated the multivariate Cox coefficients into a practical scoring system that enabled clinicians to estimate individual PFS probabilities at 180, 270 and 365 days.

### 2.6 Construction of a predictive model for G3/G4irAE and performance assessment

We constructed prediction models utilizing five machine-learning techniques: random forest, AdaBoost, Elastic-Net, Lasso, and support-vector machine (SVM). We constructed the random forest prediction model with the randomForest package (version 4.7--1.2) and the AdaBoost model with the ada package (version 2.0--5). Elastic-Net and Lasso models were fitted via the glmnet package (version 4.1--3), which unified Lasso (L1, α=1) and Elastic-Net (L1+L2, 0<α< 1) penalties within a single cyclical coordinate-descent solver and returned the entire regularization path in one pass. Hyperparameter tuning was performed objectively via the embedded K-fold cross-validation function cv.glmnet, with λ selected to minimize the mean cross-validated error, ensuring unbiased variable selection and coefficient shrinkage. Support-vector machine models were developed via the e1071 package (version 1.7--9). A model with higher area under the curve (AUC) values had better predictive value. Calibration plots were constructed via the rms package (version 6.3--0) and riskRegression (version 2025.05.20), with model reliability assessed through 1,000 bootstrap replicates to ensure performance consistency. Decision curve analysis (DCA) implemented via DCA (version 1.0), the ggDCA package (version 1.1) and dcurves (version 0.5.0) was used to quantify the model’s clinical applicability across varying risk levels. The resulting DCA plots visualized risk thresholds (x-axis) against net benefit (y-axis), where superior curve elevation denoted enhanced clinical decision-making capacity at specific risk probabilities (22).

### 2.7 Potential regulatory role of LPA in the immune microenvironment

#### 2.7.1 Data acquisition

We retrieved RNA-sequencing dataset GSE135222 from the Gene Expression Omnibus (GEO) database, which comprised tumor tissue specimens from 27 patients with advanced non-small cell lung cancer (NSCLC) treated with anti-PD-1/PD-L1 antibodies. We divided 27 patients into high and low LPA expression groups based on the mean LPA expression level.

#### 2.7.2 Differentially expressed analysis

The “limma” R package (version 3.40.6) was utilized to identify differentially expressed genes (DEGs) between the high-LPA and low-LPA expression groups, with statistical significance defined as a P value < 0.05 and an absolute log2 fold change (|log2FC|) > 1.5. These DEGs were visualized using volcano plots and heatmaps.

#### 2.7.3 Gene ontology and Kyoto encyclopedia of genes and genomes pathway enrichment analysis

Enrichment analysis for GO terms and KEGG pathways was performed using clusterProfiler (v3.14.3), with reference gene sets sourced from org.Hs.eg.db (v3.1.0) and the KEGG REST API (https://www.kegg.jp/kegg/rest/keggapi.html), respectively. Genes were mapped against these background databases under constraints of 5–5000 genes per set, with P < 0.05 considered statistically significant.

### 2.8 Statistical analysis methods

We performed statistical analyses using R software (v.4.1.3). Normally distributed continuous variables were summarized as means with standard deviations, and we compared groups using independent t tests. For variables deviating from normality, we applied Mann-Whitney U tests to evaluate group differences, reporting as medians and interquartile ranges. The description of categorical variables was performed using frequencies (percentages), with comparisons performed via the χ² test or Fisher’s Exact Test. The significance level for all two-tailed tests was set at α= 0.05.

## 3 Results

### 3.1 Patient characteristics

A total of 227 patients with complete data were included in the model development. To ensure comparability for internal validation, the dataset was randomly partitioned into training (70%) and validation (30%) sets. Baseline characteristics, treatment responses, and adverse events were similar between these data subsets (all P > 0.05), confirming successful random partitioning rather than systematic selection bias (**Table 1** and **Table 2**).

**Table 1:** Comparison of patient baseline information.

Variables	Training set (n=159)	Validation set (n=68)	Total (n=227)	Statistic value	P value
LPA	19.9 (11.7,27.65)	20.9 (12.37,28.92)	20 (11.7,27.85)	-0.438	0.661
age	62 (52,67)	62 (56,66)	62 (53,67)	-0.266	0.79
ECOG				1.595	0.207
0	80 (50.31%)	28 (41.18%)	108 (47.58%)		
1	79 (49.69%)	40 (58.82%)	119 (52.42%)		
PDL1_TPS				0.012	0.911
<1%	55 (34.59%)	23 (33.82%)	78 (34.36%)		
1%-49%	104 (65.41%)	45 (66.18%)	149 (65.64%)		
Smoking history				2.348	0.125
no	76 (47.8%)	25 (36.76%)	101 (44.49%)		
yes	83 (52.2%)	43 (63.24%)	126 (55.51%)		
BMI	24.84 (22.08,27.27)	22.95 (19.47,30.19)	24.61 (21.3,28.05)	0.873	0.383
gender				1.122	0.29
female	56 (35.22%)	29 (42.65%)	85 (37.44%)		
Male	103 (64.78%)	39 (57.35%)	142 (62.56%)		
hyperlipidemia				0.277	0.599
no	109 (68.55%)	49 (72.06%)	158 (69.6%)		
yes	50 (31.45%)	19 (27.94%)	69 (30.4%)		
Statin use				0.047	0.828
no	129 (81.13%)	56 (82.35%)	185 (81.5%)		
yes	30 (18.87%)	12 (17.65%)	42 (18.5%)		
brain metastasis				0.071	0.79
no	115 (72.33%)	48 (70.59%)	163 (71.81%)		
yes	44 (27.67%)	20 (29.41%)	64 (28.19%)		
lung metastasis				0	0.995
no	131 (82.39%)	56 (82.35%)	187 (82.38%)		
yes	28 (17.61%)	12 (17.65%)	40 (17.62%)		
liver metastasis				0.009	0.923
no	141 (88.68%)	60 (88.24%)	201 (88.55%)		
yes	18 (11.32%)	8 (11.76%)	26 (11.45%)		
bone metastasis				0.35	0.554
no	107 (67.3%)	43 (63.24%)	150 (66.08%)		
yes	52 (32.7%)	25 (36.76%)	77 (33.92%)		
adrenal gland metastasis				—	0.158*
no	145 (91.19%)	66 (97.06%)	211 (92.95%)		
yes	14 (8.81%)	2 (2.94%)	16 (7.05%)		
lymphnode metastasis				0.257	0.612
no	15 (9.43%)	5 (7.35%)	20 (8.81%)		
yes	144 (90.57%)	63 (92.65%)	207 (91.19%)		

**Table 2:** Comparison of treatment response and adverse events.

Treatment response or AE	Training set (n=159)	Validation set (n=68)	Total (n=227)	Statistic value	P value
Treatment response					
PR	91 (57.23%)	40 (58.82%)	131 (57.71%)	0.049	0.824
PD	21 (13.21%)	6 (8.82%)	27 (11.89%)	0.505	0.477
SD	47 (29.56%)	22 (32.35%)	69 (30.40%)	0.0684	0.794
G1/G2 Adverse Event	43 (27.04%)	20 (29.41%)	63 (27.75%)	0.041	0.839
Anemia	41 (25.80%)	18 (26.47%)	59 (25.99%)	0	1
Lymphocyte count decreased	40 (25.20%)	17 (25.00%)	57 (25.10%)	0	1
Neutrophil count decreased	40 (25.20%)	18 (26.50%)	58 (25.60%)	0	0.97
White blood cell count decreased	40 (25.20%)	16 (23.50%)	56 (24.70%)	0.01	0.93
Vomiting	33 (20.80%)	12 (17.60%)	45 (19.80%)	0.13	0.72
Fatigue	29 (18.20%)	13 (19.10%)	42 (18.50%)	0	1
Platelet count decreased	26 (16.40%)	14 (20.60%)	40 (17.60%)	0.33	0.56
Blood creatinine increased	20 (12.60%)	9 (13.20%)	29 (12.80%)	0	1
Hypothyroidism	20 (12.60%)	8 (11.80%)	28 (12.30%)	0	1
Aspartate aminotransferase increased	18 (11.30%)	7 (10.30%)	25 (11.00%)	0	1
Blood bilirubin increased	18 (11.30%)	6 (8.80%)	24 (10.60%)	0.11	0.74
Alanine aminotransferase increased	17 (10.70%)	8 (11.80%)	25 (11.00%)	0	1
Rash	17 (10.70%)	10 (14.70%)	27 (11.90%)	0.4	0.53
Gamma-glutamyltransferase increased	10 (6.30%)	4 (5.90%)	14 (6.20%)	—	1*
Hyperthyroidism	10 (6.30%)	6 (8.80%)	16 (7.00%)	—	0.57*
Constipation	6 (3.80%)	3 (4.40%)	9 (4.00%)	—	1*
Interstitial lung disease	3 (1.90%)	0 (0.00%)	3 (1.30%)	—	0.56*
Myocarditis	1 (0.60%)	1 (1.50%)	2 (0.90%)	—	0.51*
G3/G4 Adverse Event	28 (17.61%)	14 (20.59%)	42 (18.50%)	0.117	0.732
Lymphocyte count decreased	20 (12.58%)	11 (16.18%)	31 (13.66%)	0.26	0.61
Platelet count decreased	17 (10.69%)	10 (14.71%)	27 (11.89%)	0.4	0.53
Neutrophil count decreased	16 (10.06%)	9 (13.24%)	25 (11.01%)	0.22	0.64
Anemia	24 (15.09%)	10 (14.71%)	34 (14.98%)	0	1
White blood cell count decreased	19 (11.95%)	9 (13.24%)	28 (12.33%)	0	0.96
Aspartate aminotransferase increased	14 (8.81%)	5 (7.35%)	19 (8.37%)	0.01	0.92
Alanine aminotransferase increased	16 (10.06%)	6 (8.82%)	22 (9.69%)	0	0.96
Constipation	2 (1.26%)	1 (1.47%)	3 (1.32%)	—	1*
Fatigue	13 (8.18%)	7 (10.29%)	20 (8.81%)	—	0.79*
Vomiting	10 (6.29%)	5 (7.35%)	15 (6.61%)	—	0.77*
Hypothyroidism	6 (3.77%)	4 (5.88%)	10 (4.41%)	—	0.49*
Hyperthyroidism	7 (4.40%)	2 (2.94%)	9 (3.96%)	—	0.73*
Rash	3 (1.89%)	2 (2.94%)	5 (2.20%)	—	0.64*
Gamma-glutamyltransferase increased	3 (1.89%)	1 (1.47%)	4 (1.76%)	—	1*
Blood bilirubin increased	8 (5.03%)	3 (4.41%)	11 (4.85%)	—	1*
Blood creatinine increased	9 (5.66%)	4 (5.88%)	13 (5.73%)	—	1*
Interstitial lung disease	8 (5.03%)	1 (1.47%)	9 (3.96%)	—	0.29*
Myocarditis	1 (0.60%)	1 (1.50%)	2 (0.90%)	—	0.51*

Items marked with an asterisk (*) were analyzed using Fisher’s exact test, with only P values reported for the statistical results.

### 3.2 A prognostic model was constructed to predict PFS in patients receiving first-line chemoimmunotherapy

**Figure 2:**
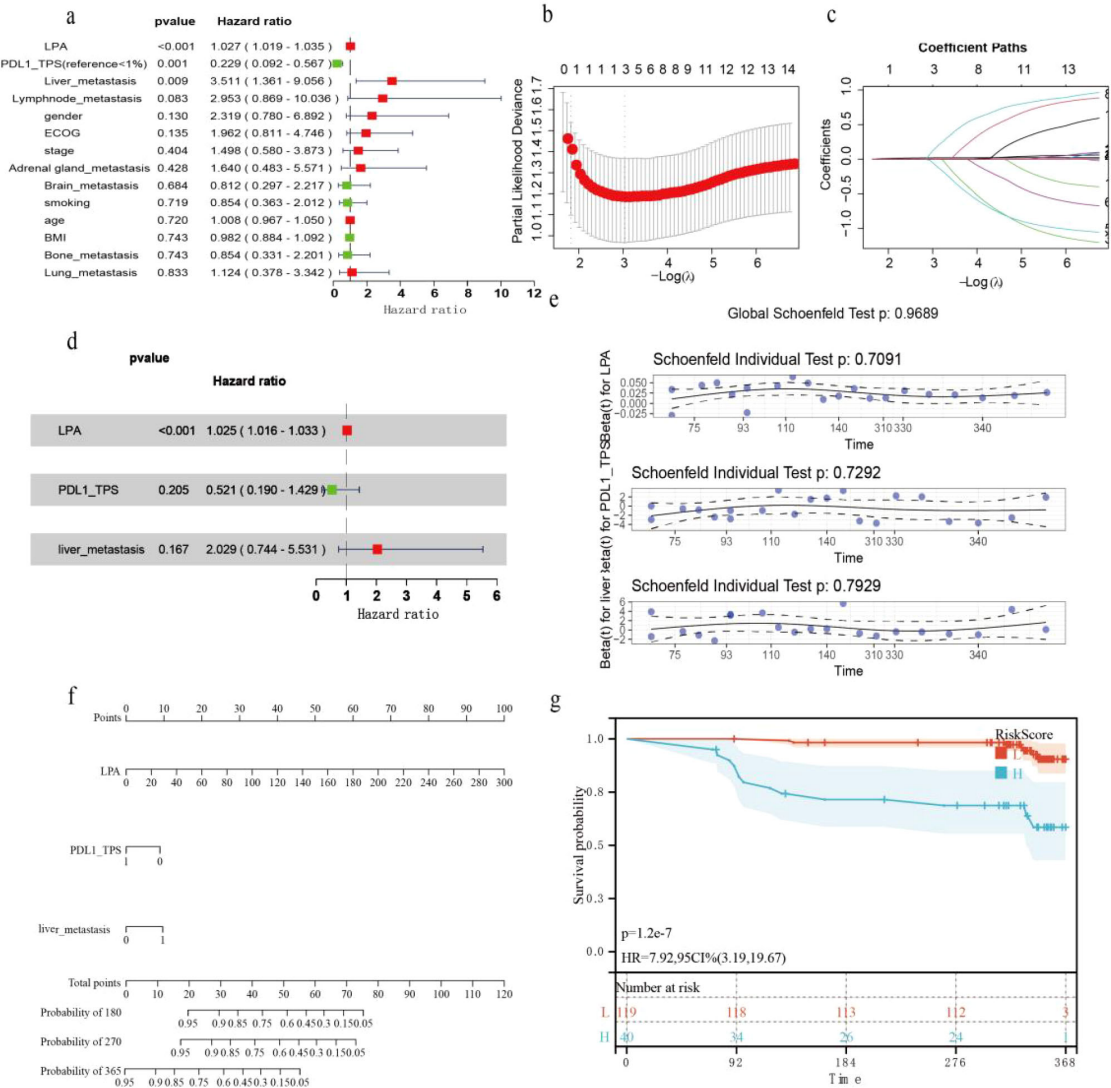
Illustrated the development of a prognostic model. **(a)** Forest plot demonstrating the prognostic value of all the indicators in univariate Cox regression analysis. **(b)** Regularization path plot. **(c)** Cross-validation curve plot. **(d)** Forest plot demonstrating the prognostic indicators in multivariate Cox regression analysis. **(e)** Nomogram for this model. **(f)** Global Schoenfeld test for multivariate Cox regression. **(g)** K–M curve for the prognostic model score.

Univariate Cox regression analysis revealed that the serum LPA concentration and liver metastasis were risk factors for PFS, whereas a PD-L1 TPS of 1%-49% was associated with a better PFS than TPS <1% (**Figure 2a**). The cross-validation curve plot indicated that partial likelihood deviance was minimized with 3 variables retained (**Figure 2b**). The regularization path plot illustrated the trajectories of individual variable coefficients across a spectrum of λ values (**Figure 2c**). The minimum lambda value was 0.04809. Ultimately, only LPA (coefficient=0.0204), PDL1_TPS (coefficient=-0.0721), and liver_metastasis (coefficient=0.1444) retained nonzero coefficients. Multivariate Cox regression analysis identified elevated LPA as an adverse prognostic factor for PFS (**Figure 2d**). We built a nomogram to visualize the prognostic model (**Figure 2e**). We applied the Schoenfeld individual test to the multivariable Cox model. All covariates fulfilled the proportional hazards (PH) assumption. The global Schoenfeld test returned P = 0.9689> 0.05, confirming that the overall model fulfilled the proportional hazards assumption (**Figure 2f**). The risk score formula for this model was as follows: score=0.0262× LPA-1.475×PDL1_TPS+1.256×liver_metastasis. The optimal risk score cutoff was determined via the R package maxstat (version 0.7--25). The resulting cutoff value was 0.515. Patients whose risk score exceeded the cutoff score had an HR of 7.92 (P < 0.001) (**Figure 2g**).

### 3.3 Model performance evaluation

**Figure 3:**
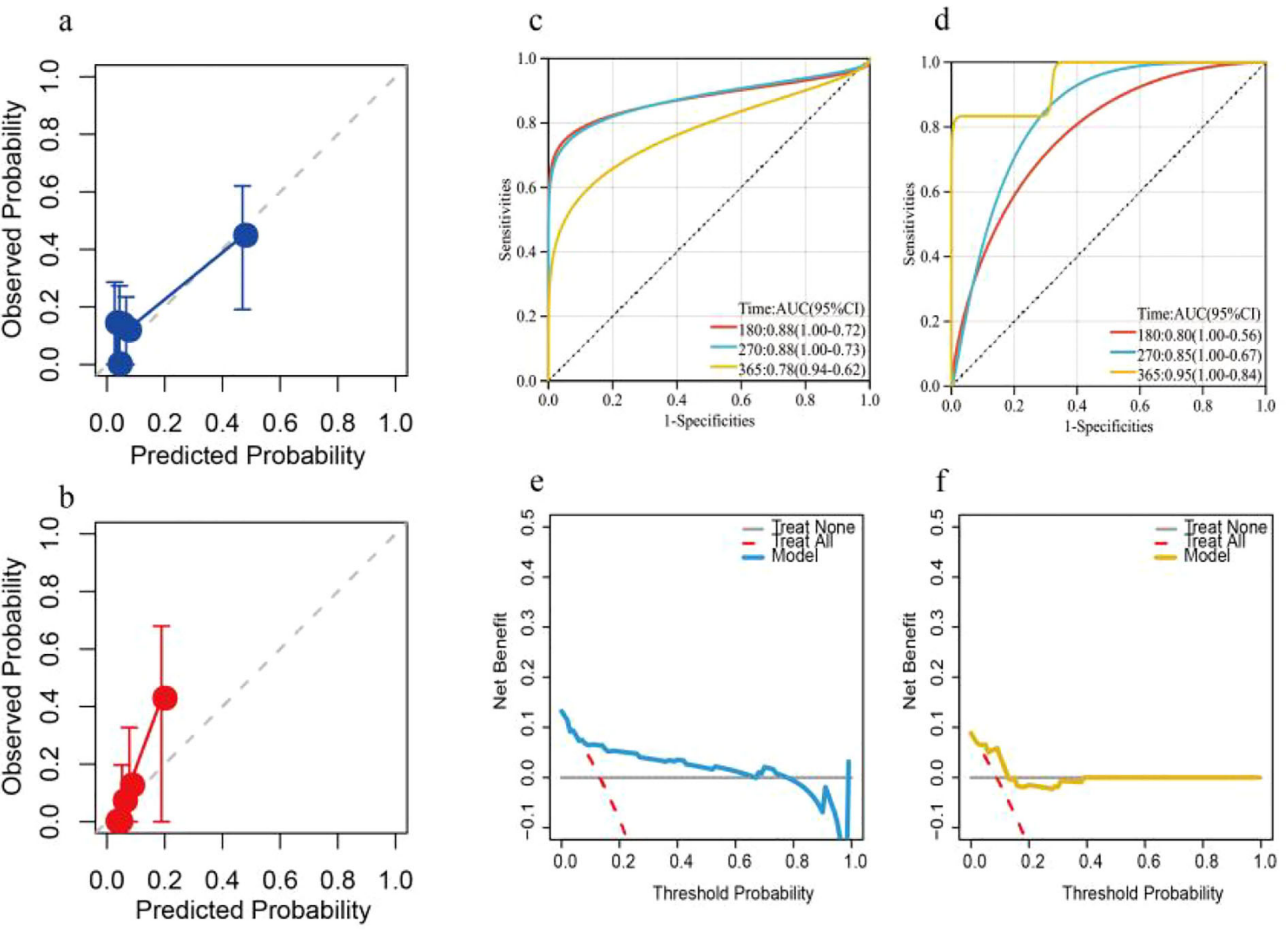
Showed the model’s optimal performance in the two datasets [**(a, b)** The calibration curve revealed strong agreement in the training and validation sets; **(c, d)** The ROC curve revealed the predictive ability in the training and validation sets; **(e, f)**. The DCA curve revealed a clinical benefit in the training set and validation set].

The C-index was 0.816 (95% CI: 0.695 - 0.937) and 0.832 (95% CI: 0.692 - 0.973) in the two sets. The predicted and observed 1-year PFS probabilities in the training dataset were in strong agreement, as demonstrated by calibration plots in the training (**Figure 3a**) and external validation (**Figure 3b**) datasets, highlighting good agreement between predicted and observed probabilities. The AUC values for the model were presented in **Figure 3c** and 3d. Decision curve analysis further quantified the net clinical benefit across a spectrum of risk thresholds (**Figures 3e, f**), confirming the practical utility of the nomogram in guiding therapeutic decisions for patients in both cohorts.

### 3.4 Adrenal gland metastasis might influence the relationship between LPA and shortened progression-free survival

**Figure 4:**
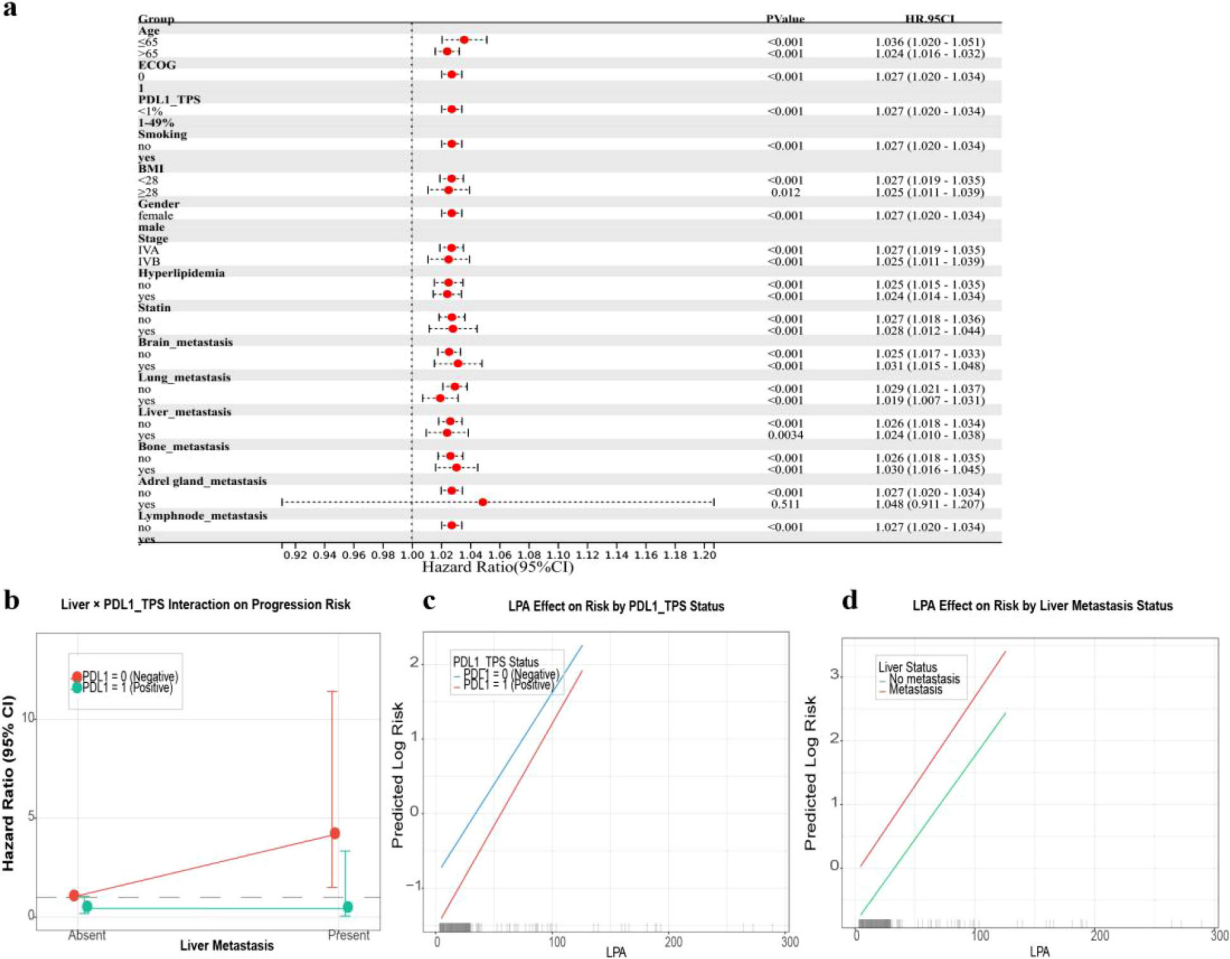
Adrenal gland metastasis might influence the relationship between LPA and shortened progression-free survival. [**(a)** Subgroup analysis forest plot for LPA; **(b)** interaction effect diagram for PDL1_TPS and liver_metastasis; **(c)** Interaction effect diagram for PDL1_TPS and LPA; **(d)** Interaction effect diagram for LPA and liver_metastasis].

There was no evidence in subgroup analyses suggesting that age, BMI, cancer stage, hyperlipidemia status, statin use or brain, liver, lung, bone metastasis influenced the relationship between LPA and PFS. However, in the subgroup of patients with adrenal metastasis, we did not find a significant statistical correlation between LPA and shorter PFS (**Figure 4a**). For several subgroups, HR estimation was precluded by complete separation (i.e., no progression events occurred in one of the comparator groups), resulting in non-convergent phenomenon. Consequently, these entries were left blank in the forest plot to indicate unestimable rather than missing estimates. As shown in **Figures 4b-d**, in the multivariable Cox regression model, no pairwise interaction effects were found among any of the predictors in our prognostic model.

### 3.5 Development of a machine learning model for predicting G3/4 AEs

**Figure 5:**
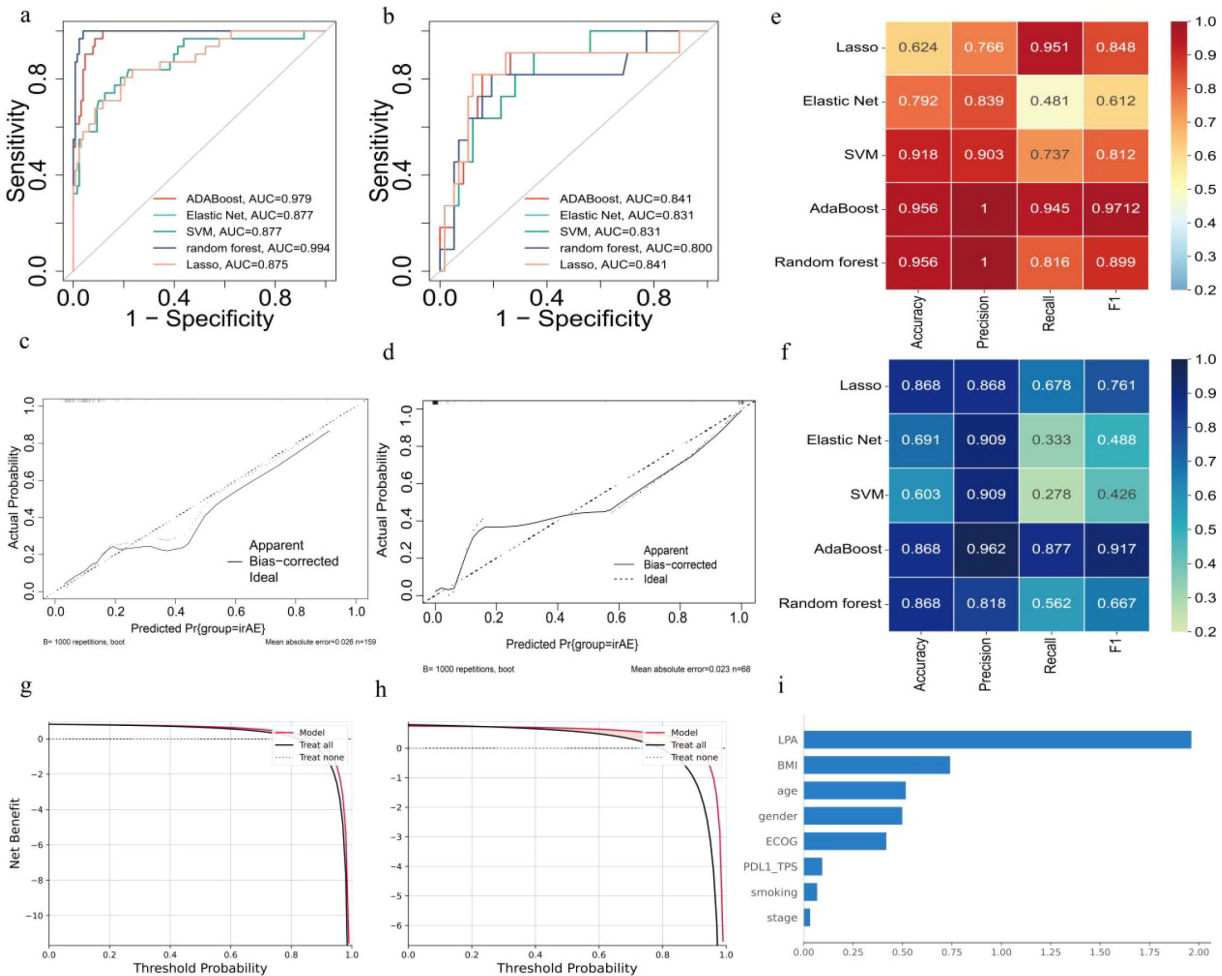
demonstrated the AdaBoost model’s effectiveness in predicting G3/4 AEs. **(a, b)** ROC curves for the development and validation sets; **(c, d)** calibration plots for the development and validation sets; **(e, f)** a heatmap indicating AdaBoost’s superior accuracy, precision, recall, and F1 score in the development and validation sets; **(g, h)** DCA curve for the development and validation sets; and **(i)** a SHAP bar chart.

Forty-two patients experienced G3/4 AEs. The random forest model showed the highest AUC value in the development set (**Figure 5a**); however, AdaBoost outperformed it in the validation set, with the highest AUC value (**Figure 5b**). The calibration plots in **Figure 5c, d** demonstrated a strong alignment between the predicted and actual probabilities of Grade 3/4 AEs in the AdaBoost model. The AdaBoost model outperformed the other methods across four metrics, including accuracy, precision, recall, and F1 score on both the development and validation sets (**Figures 5e, f**), leading to its selection for constructing the predictive model. We found that the AdaBoost model provided substantial net clinical benefits in both datasets (**Figures 5g, h**). The SHapley Additive exPlanations (SHAP) results for AdaBoost showed that LPA had the highest contribution to the model, followed by BMI and age (**Figure 5i**).

### 3.6 Validation of the Cox regression model in the per-protocol population undergoing initial chemoimmunotherapy

**Figure 6:**
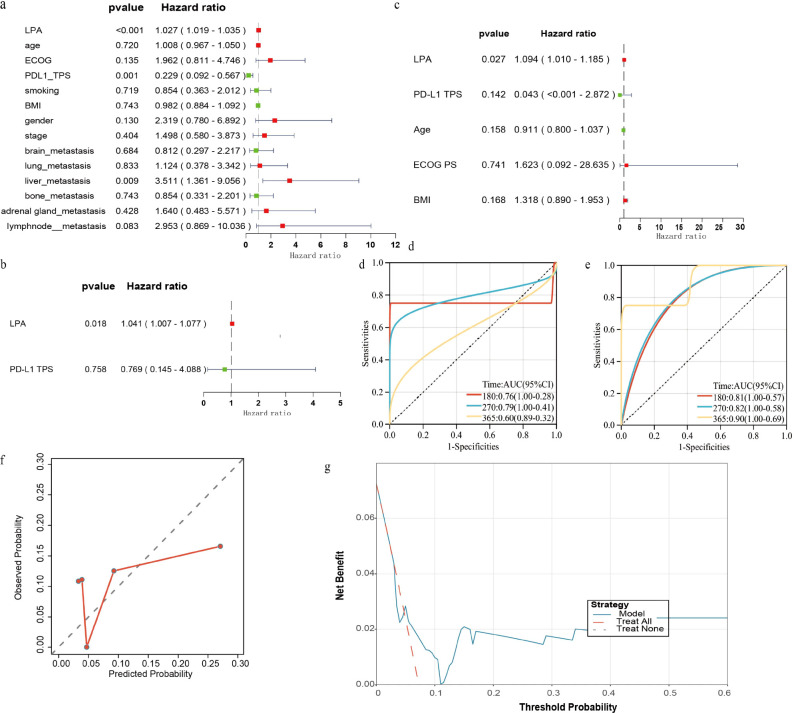
The prognostic efficacy of the Cox regression model was successfully validated in the per-protocol population. **(a, b)** LPA score were identified as independent risk factors in the per-protocol population of the training set. **(c)** Elevated LPA was an adverse prognostic factor for PFS in the per-protocol population of the validation set. **(d, e)** The model demonstrated good prognostic value in both the development and validation sets of the per-protocol population. **(f)** The predicted probabilities of the model were in good agreement with the actual probabilities. **(g)** The model conferred clinical benefit in the development set of the per-protocol population.

Finally, a total of 124 and 53 per-protocol patients received the assigned treatment and completed all follow-up in the two sets, respectively. In the per-protocol population of the training set, the serum LPA concentration remained an independent risk factor for progression-free survival (**Figures 6a, b**). In the per-protocol population of the validation set, only 6 patients experienced disease progression. Due to this limited number of events, we could only estimate HR values and 95%CIs for 5 variables (LPA, PD-L1 TPS, Age, ECOG PS, and BMI); the remaining variables suffered from complete separation and thus had undefined or infinitely large HR estimates. LPA was still found as a risk factor for PFS in the validation set (**Figure 6c**). The model’s prognostic performance was additionally validated within the per-protocol population. The AUC values for the model are presented in **Figures 6d, e**. The predicted and actual 1-year PFS probabilities aligned closely in the per-protocol populations, as demonstrated by calibration plots in training set (**Figure 6f**). We assessed the net clinical benefit across a spectrum of risk thresholds in the training set (**Figure 6g**). Given the limited number of events in the validation cohort (n=6), calibration curves and decision curve analysis were not performed to avoid unreliable estimates and overfitting.

### 3.6 Differential expression analysis and enrichment analysis revealed that LPA might down-regulate PD-L1 expression and PD-1 checkpoint pathway by inhibiting LTA and CD28

**Figure 7:**
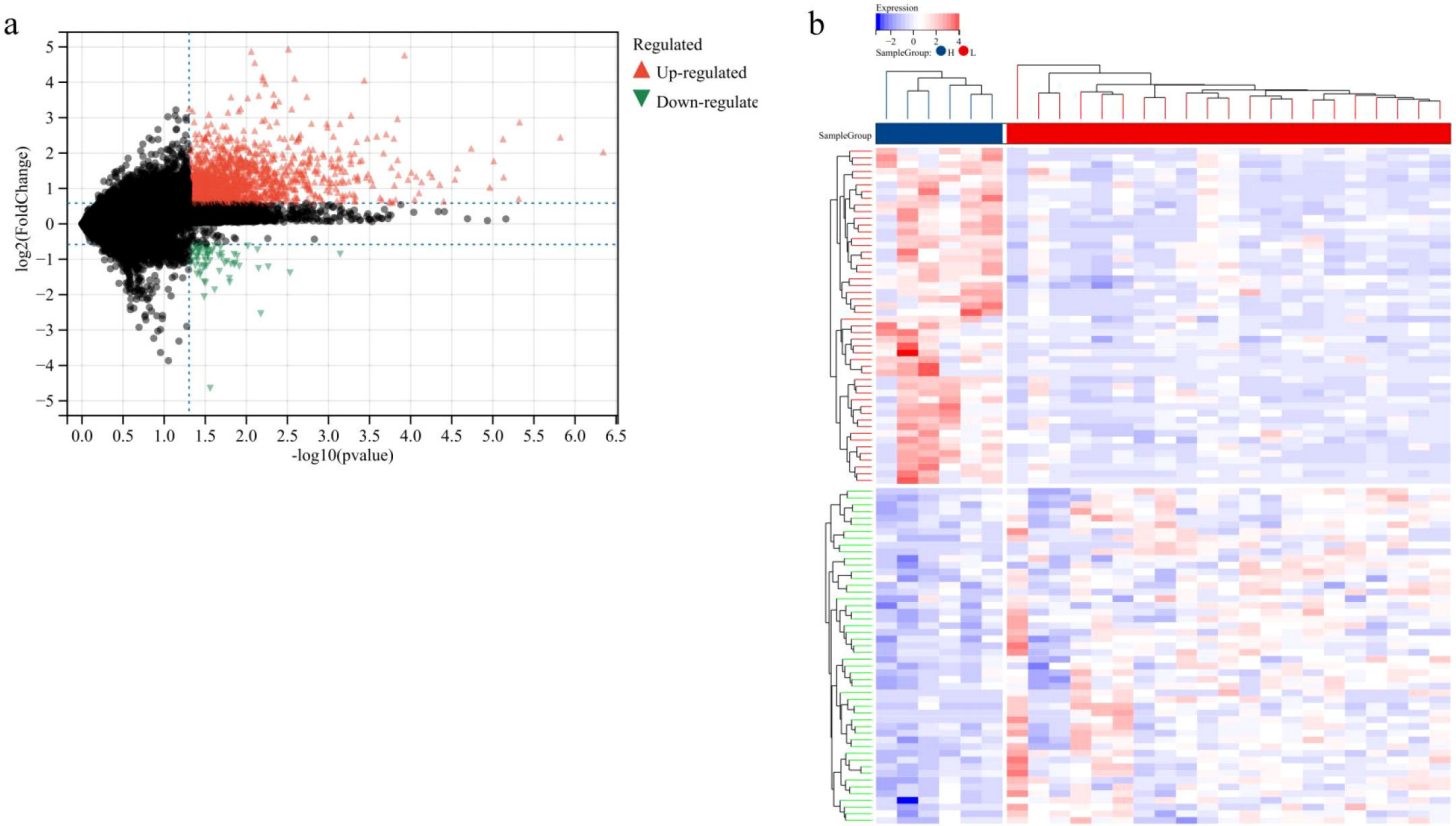
Differential expression analysis results [**(a)** volcano plot; **(b)** heatmap].

We divided the samples in GSE135222 into LPA high/low expression groups based on the mean expression value of LPA. Differential expression analysis was performed using the LIMMA package, and a total of 1439 genes were significantly up-regulated and 68 genes were significantly down-regulated (**Figures 7a, b**).

**Figure 8:**
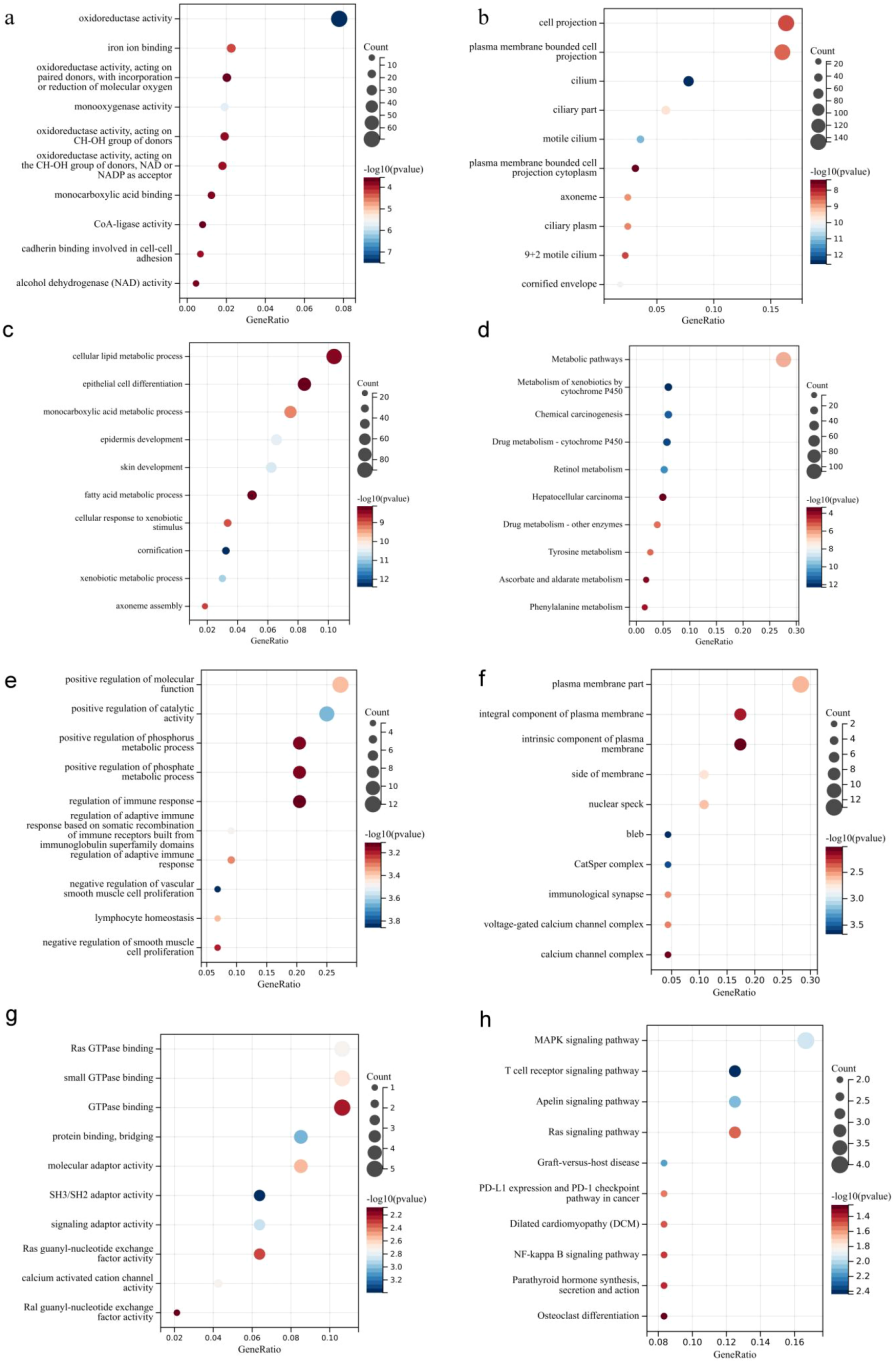
GO and KEGG analysis [**(a)** biological processes for upregulated genes; **(b)** cellular components for upregulated genes; **(c)** molecular functions for upregulated genes; **(d)** KEGG for upregulated genes; **(e)** Biological processes for downregulated genes; **(f)** cellular components for downregulated genes **(g)** molecular functions for down-regulated genes **(h)** KEGG for down-regulated genes].

In the LPA high expression group, the upregulated differentially expressed genes were significantly enriched in the biological processes of monocarboxylic acid metabolic process, cellular response to xenobiotic stimulus, cellular lipid metabolic process, fatty acid metabolic process (**Figure 8a**), cellular components of cilium, motile cilium, axoneme, ciliary plasm, plasma membrane (**Figure 8b**) and molecular function of oxidoreductase activity, monooxygenase activity, iron ion binding and monocarboxylic acid binding (**Figure 8c**). Moreover, the upregulated differentially expressed genes are significantly enriched in the metabolism of xenobiotics by cytochrome P450, chemical carcinogenesis, retinol metabolism, metabolic pathways, drug metabolism - other enzymes signaling pathway (**Figure 8d**).

The significantly downregulated genes in the high LPA expression group were enriched in the biological processes of regulation of adaptive immune response based on somatic recombination of immune, lymphocyte homeostasis, regulation of adaptive immune responses(**Figure 8e**), the cellular components of plasma membrane part, CatSper complex, side of membrane, plasma membrane part, integral component of plasma membrane, intrinsic component of plasma membrane(**Figure 8f**), and the molecular function of SH3/SH2 adaptor activity, protein binding, bridging, signaling adaptor activity, calcium activated cation channel activity, Ras GTPase binding, small GTPase binding, molecular adaptor activity(**Figure 8g**). In terms of KEGG, significantly downregulated genes in the high LPA expression group were enriched in T cell receptor signaling pathway and PD-L1 expression and PD-1 checkpoint pathway in cancer signaling pathway (**Figure 8h**). Among them, lymphotoxin alpha(LTA) and CD28 was found down-regulated and enriched in the PD-L1 expression and PD-1 checkpoint pathway in cancer signaling pathway.

## 4 Discussion

In a cohort of 380 advanced NSCLC patients undergoing first-line chemoimmunotherapy, Giuseppe L. Banna et al. established the NHS-Lung score to prognosticate OS and PFS. The model incorporates the number of metastatic sites, histological subtype, and SII. C-index values of 0.623 and 0.613 were achieved for OS and PFS, respectively (23). Their study enrolled patients across the full spectrum of PD-L1 expression (TPS >1%, 1-49%, and ≥50%). However, the TPS <50% subgroup receiving first-line chemoimmunotherapy represents the population with the greatest unmet need for predictive modeling. Unlike high-expression (TPS ≥50%) patients who might be candidates for immunotherapy monotherapy, this subgroup universally required combination therapy, facing distinct toxicity profiles and greater prognostic uncertainty that necessitate precise predictive modeling. To the best of our knowledge, this model is the first to address PFS in metastatic driver gene-negative LUAD patients with a PD-L1 TPS<50% who were receiving first-line chemoimmunotherapy. LASSO regression offered the advantages of addressing multicollinearity issues and preventing overfitting. Using LASSO regression, we identified three prognostic factors: LPA, liver metastasis, and PD-L1 TPS. Multivariate Cox regression was employed to create a prognostic model for training-set patients who completed follow-up, which was validated in the validation set of patients who completed follow-up and in the per-protocol populations of both datasets. Clinicians could use the nomogram to quickly estimate the 180-, 270-, and 365-day PFS probabilities for individual patients.

Currently, predictive models for treatment-related adverse events in patients with advanced NSCLC have been largely limited to immunotherapy monotherapy. For instance, Qing Qiu et al. conducted a cohort study of 357 NSCLC patients receiving PD-1/PD-L1 inhibitor therapy, developing a model to predict immune-related adverse events based on the SII,BMI, and age (24). Agnish Dey et al. conducted a cohort study of 617 patients with metastatic NSCLC treated with immunotherapy, developing a predictive model for adverse events using a random forest classification algorithm based on baseline characteristics (25). However, predictive models for adverse events associated with first-line chemoimmunotherapy in metastatic lung adenocarcinoma remain lacking. In this study, we constructed a predictive model for G3/4 adverse events via the AdaBoost method, which performed the best in both the training and validation sets. Accurate toxicity probability data can be integrated into decision aids, allowing patients to balance survival gain versus quality-of-life impairment, and enabling clinicians to formulate individualized adverse event monitoring strategies.

Elevated serum LPA level was independent risk factor for shorter PFS. Although primarily determined by genetic factors, LPA levels might also be influenced by sex hormones, age, and comorbidities (2, 26–28). In subgroup analyses, significant interaction with LPA was observed only for adrenal metastasis, while no significant effect modification was found for other variables. No drugs have been approved specifically for reducing LPA levels, and alirocumab has been found to decrease lipoprotein(a) levels by 10–30% (29). In this study, no patients received evolocumab, alirocumab, or inclisiran before or during follow-up. Although no statins have been reported to regulate serum LPA levels, we still performed subgroup analyses according to statin use and baseline hyperlipidemia and found that neither factor significantly altered the relationship between elevated LPA levels and shorter PFS.

In this study, differential expression analysis revealed that CD28 and LTA were significantly downregulated in the high LPA-expression group. Lymphotoxin-alpha (LTα), recognized as LT-alpha or LTA, functions as an inflammatory cytokine with a key impact on the regulation and persistence of inflammatory responses (30). LTα was essential for the formation of secondary lymphoid organs, as it paired with LTβ to create heterotrimeric complexes such as LTα1β2 and LTα2β1. These complexes facilitated communication between lymphocytes and nearby fibroblasts, as well as with epithelial and myeloid cells that carried the LTβ receptor (31). The binding of B7–1 or B7–2 proteins on the surface of antigen-presenting cells to the CD28 receptor on T cells provided the necessary co-stimulatory signals for T cell activation. The expression of CD28 was also necessary for CD8+T cells to respond to PD-1 monoclonal antibodies (32). Previous studies had shown that, following anti-PD-1 therapy, the circulating CD8^+^T cells in lung-cancer patients were predominantly CD28-positive and exhibited vigorous proliferation (33). After binding with its ligand, PD-1 could counteract the signaling of TCR and CD28, thereby inhibiting the activation and proliferation of T cells (34, 35). Therefore, inhibiting the expression of CD28 and LTA could inhibit the differentiation, activation, and maturation of CD8+T cells, thereby playing a key role in mediating primary drug resistance to PD-1/PD-L1 monoclonal antibodies.

Our model could assist in identifying individuals who are likely to progress within a year during first-line chemoimmunotherapy. The phase III TASUKI-52 trial demonstrated that first-line treatment with nivolumab combined with platinum-based chemotherapy and bevacizumab enhanced PFS and OS in patients with advanced or recurrent NSCLC without driver gene mutations (12, 36). Thus, an alternative initial treatment is available for patients who are unlikely to benefit from first-line chemoimmunotherapy. Whether these patients would gain greater benefit from chemotherapy combined with immunotherapy plus antiangiogenic therapy needs further investigated.

This study had several limitations. The model’s generalizability might be limited, as all participants in our prospective cohort study were Chinese. Second, as this was a single-center prospective cohort study, the representativeness and external validity of the sample needed further investigation through multicenter studies. A limitation regarding the mechanistic interpretation of LPA signaling was that the publicly available transcriptomic datasets (GSE135222) were derived from tumor samples of patients receiving immune checkpoint inhibitor monotherapy, rather than from first-line platinum-based chemotherapy plus immunotherapy combination. Chemotherapy- induced effects might not have been fully captured by monotherapy-derived transcriptomic profiles. We will investigate the regulatory effect and mechanism of LPA on lung adenocarcinoma model mice treated with chemoimmunotherapy through experiments in the future.

## 5 Conclusion

Elevated serum LPA level was independent risk factor for shorter PFS. We developed models for PFS and G3/4 AEs in patients with metastatic driver gene-negative lung adenocarcinoma who were receiving first-line chemoimmunotherapy. These models can aid in the early identification of patients who are less likely to benefit from this treatment regimen.

## Data Availability

The original contributions presented in the study are included in the article/supplementary material. Further inquiries can be directed to the corresponding author.

## References

[1] Lippi G Franchini M Salvagno GL Guidi GC . Lipoprotein[a] and cancer: anti-neoplastic effect besides its cardiovascular potency. Cancer Treat Rev. (2007) 33:427–36. doi: 10.1016/j.ctrv.2007.02.006. PMID: 17442497

[2] Duarte Lau F Giugliano RP . Lipoprotein(a) and its significance in cardiovascular disease: a review. JAMA Cardiol. (2022) 7:761–9. doi: 10.1001/jamacardio.2022.0987. PMID: 35583875

[3] Yang H Chen X Hu W Lv D Ding W Tang L . Lipoprotein(a) level and its association with tumor stage in male patients with primary lung cancer. Clin Chem Lab Med. (2009) 47:452–7. doi: 10.1515/CCLM.2009.094. PMID: 19222374

[4] Sun D Huang T Li J Liu M Zhang X Cui M . Development and validation of a predictive model for anticipatory grief in family caregivers of cancer patients: based on LASSO-logistic regression model. Psychooncology. (2025) 34:e70236. doi: 10.1002/pon.70236. PMID: 40670296

[5] Khwaja B Thankam FG Agrawal DK . Mitochondrial DAMPs and altered mitochondrial dynamics in OxLDL burden in atherosclerosis. Mol Cell Biochem. (2021) 476:1915–28. doi: 10.1007/s11010-021-04061-0. PMID: 33492610

[6] Zhou C Weng J Liu C Liu S Hu Z Xie X . Disruption of SLFN11 deficiency–induced CCL2 signaling and macrophage M2 polarization potentiates anti–PD-1 therapy efficacy in hepatocellular carcinoma. Gastroenterology. (2023) 164:1261–78. doi: 10.1053/j.gastro.2023.02.005. PMID: 36863689

[7] Scipione CA Sayegh SE Romagnuolo R Tsimikas S Marcovina SM Boffa MB . Mechanistic insights into lp(a)-induced IL-8 expression: A role for oxidized phospholipid modification of apo(a). J Lipid Res. (2015) 56:2273–85. doi: 10.1194/jlr.M060210. PMID: 26474593 PMC4655984

[8] Wiesner P Tafelmeier M Chittka D Choi S-H Zhang L Byun YS . MCP-1 binds to oxidized LDL and is carried by lipoprotein(a) in human plasma. J Lipid Res. (2013) 54:1877–83. doi: 10.1194/jlr.M036343. PMID: 23667177 PMC3679389

[9] Yang X Luo B Tian J Wang Y Lu X Ni J . Biomarkers and ImmuneScores in lung cancer: predictive insights for immunotherapy and combination treatment strategies. Biol Proced Online. (2025) 27:25. doi: 10.1186/s12575-025-00287-0. PMID: 40640703 PMC12243280

[10] Zhou C Chen G Huang Y Zhou J Lin L Feng J . Camrelizumab plus carboplatin and pemetrexed as first-line therapy for advanced non-squamous non-small-cell lung cancer: 5-year outcomes of the CameL randomized phase 3 study. J Immunother Cancer. (2024) 12:e009240. doi: 10.1136/jitc-2024-009240. PMID: 39608979 PMC11603811

[11] Se B Eysa A Karim N . Clinical insights: five-year follow-up of KEYNOTE-189 trial outcomes and more. Transl Lung Cancer Res. (2024) 13:2095–7. doi: 10.21037/tlcr-24-198. PMID: 39263022 PMC11384474

[12] De Castro G Kudaba I Wu Y-L Lopes G Kowalski DM Turna HZ . Five-year outcomes with pembrolizumab versus chemotherapy as first-line therapy in patients with non–small-cell lung cancer and programmed death ligand-1 tumor proportion score ≥ 1% in the KEYNOTE-042 study. J Clin Oncol. (2023) 41:1986–91. doi: 10.1200/JCO.21.02885. PMID: 36306479 PMC10082298

[13] Lee KH Lee J-S Sugawara S Kang JH Kim HR Inui N . First-line nivolumab plus platinum chemotherapy and bevacizumab for advanced nonsquamous non-small cell lung cancer: a 3-year follow-up of the phase 3 randomized TASUKI-52 trial. Lung Cancer. (2025) 201:108109. doi: 10.1016/j.lungcan.2025.108109. PMID: 39893774

[14] Gandhi L Rodríguez-Abreu D Gadgeel S Esteban E Felip E De Angelis F . Pembrolizumab plus chemotherapy in metastatic non-small-cell lung mancer. N Engl J Med. (2018) 378(22):2078–92. doi: 10.1056/NEJMoa1801005. PMID: 29658856

[15] Garassino MC Gadgeel S Speranza G Felip E Esteban E Dómine M . Pembrolizumab plus pemetrexed and platinum in nonsquamous non–small-cell lung cancer: 5-year outcomes from the phase 3 KEYNOTE-189 study. J Clin Oncol. (2023) 41:1992–8. doi: 10.1200/JCO.22.01989. PMID: 36809080 PMC10082311

[16] Gadgeel S Rodríguez-Abreu D Speranza G Esteban E Felip E Dómine M . Updated analysis from KEYNOTE-189: Pembrolizumab or placebo plus pemetrexed and platinum for previously untreated metastatic nonsquamous non–small-cell lung cancer. J Clin Oncol. (2020) 38:1505–17. doi: 10.1200/JCO.19.03136. PMID: 32150489

[17] Zhang X Wu M Chen J Zheng K Du H Li B . Comparative efficacy of immune checkpoint inhibitors combined with chemotherapy in patients with advanced driver-gene negative non-small cell lung cancer: a systematic review and network meta-analysis. Heliyon. (2024) 10:e30809. doi: 10.1016/j.heliyon.2024.e30809. PMID: 38774326 PMC11107224

[18] Brunetti L Santo V Galletti A Gelibter A Lugini A Spinelli GP . TTF-1 negativity predicts poor outcomes in advanced non-squamous NSCLC also in the immunotherapy era: a multicenter cohort study and meta-analysis. Cancers. (2025) 17:2188. doi: 10.3390/cancers17132188. PMID: 40647486 PMC12248803

[19] Zhou C Chen G Huang Y Zhou J Lin L Feng J . Camrelizumab plus carboplatin and pemetrexed versus chemotherapy alone in chemotherapy-naive patients with advanced non-squamous non-small-cell lung cancer (CameL): a randomised, open-label, multicentre, phase 3 trial. Lancet Respir Med. (2021) 9:305–14. doi: 10.1016/S2213-2600(20)30365-9. PMID: 33347829

[20] Makris UE Alvarez CA Mortensen EM Mansi IA . Association of statin use with increased risk of musculoskeletal conditions: A retrospective cohort study. Drug Saf. (2018) 41:939–50. doi: 10.1007/s40264-018-0682-y. PMID: 29797239 PMC6143406

[21] Wu Y-L Planchard D Lu S Sun H Yamamoto N Kim D-W . Pan-asian adapted clinical practice guidelines for the management of patients with metastatic non-small-cell lung cancer: A CSCO–ESMO initiative endorsed by JSMO, KSMO, MOS, SSO and TOS. Ann Oncol. (2019) 30:171–210. doi: 10.1093/annonc/mdy554. PMID: 30596843

[22] Chi Y Ma G Liu Q Xiang Y Liu D Du J . Multi-omics analysis reveals glutathione metabolism-related immune suppression and constructs a prognostic model in lung adenocarcinoma. Front Immunol. (2025) 16:1608407. doi: 10.3389/fimmu.2025.1608407. PMID: 40672941 PMC12263636

[23] Banna GL Cantale O Muthuramalingam S Cave J Comins C Cortellini A . Efficacy outcomes and prognostic factors from real-world patients with advanced non-small-cell lung cancer treated with first-line chemoimmunotherapy: the spinnaker retrospective study. Int Immunopharmacol. (2022) 110:108985. doi: 10.1016/j.intimp.2022.108985. PMID: 35777264

[24] Qiu Q Wu C Tang W Ji L Dai G Gao Y . Development and validation of a risk-prediction model for immune-related adverse events in patients with non-small-cell lung cancer receiving PD-1/PD-L1 inhibitors. J Zhejiang Univ-Sci B. (2023) 24:935–42. doi: 10.1631/jzus.B2200631. PMID: 37752094 PMC10522565

[25] Dey A Austin M Kluger HM Trunova N Mann H Shire N . Association between immune-mediated adverse events and efficacy in metastatic non-small-cell lung cancer patients treated with durvalumab and tremelimumab. Front Immunol. (2022) 13:1026964. doi: 10.3389/fimmu.2022.1026964. PMID: 36405729 PMC9670978

[26] Nordestgaard BG Langsted A . Lipoprotein(a) and cardiovascular disease. Lancet. (2024) 404:1255–64. doi: 10.1016/S0140-6736(24)01308-4. PMID: 39278229

[27] Lampsas S Xenou M Oikonomou E Pantelidis P Lysandrou A Sarantos S . Lipoprotein(a) in atherosclerotic diseases: from pathophysiology to diagnosis and treatment. Molecules. (2023) 28:969. doi: 10.3390/molecules28030969. PMID: 36770634 PMC9918959

[28] Xu R Wang Z Dong J Yu M Zhou Y . Lipoprotein(a) and panvascular disease. Lipids Health Dis. (2025) 24:186. doi: 10.1186/s12944-025-02600-y. PMID: 40413492 PMC12103022

[29] O’Donoghue ML Fazio S Giugliano RP Stroes ESG Kanevsky E Gouni-Berthold I . Lipoprotein(a), PCSK9 inhibition, and cardiovascular risk: insights from the FOURIER trial. Circulation. (2019) 139:1483–92. doi: 10.1161/CIRCULATIONAHA.118.037184. PMID: 30586750

[30] Pissetti CW De Oliveira RF Correia D Nascentes GAN Llaguno MM Rodrigues V . Association between the lymphotoxin-alpha gene polymorphism and chagasic cardiopathy. J Interferon Cytokine Res. (2013) 33:131–5. doi: 10.1089/jir.2012.0024. PMID: 23289732

[31] Hagge DA Saunders BM Ebenezer GJ Ray NA Marks VT Britton WJ . Lymphotoxin-α and TNF have essential but independent roles in the evolution of the granulomatous response in experimental leprosy. Am J Pathol. (2009) 174:1379–89. doi: 10.2353/ajpath.2009.080550. PMID: 19246648 PMC2671369

[32] Wang PH Washburn RS Mariuzza DL Lin W-H Gill AL Ahmed R . Reciprocal transmission of activating and inhibitory signals and cell fate in regenerating T cells. Cell Rep. (2023) 42:113155. doi: 10.1016/j.celrep.2023.113155. PMID: 37756164 PMC10872930

[33] Kamphorst AO Wieland A Nasti T Yang S Zhang R Barber DL . Rescue of exhausted CD8 T cells by PD-1–targeted therapies is CD28-dependent. Science. (2017) 355:1423–7. doi: 10.1126/science.aaf0683. PMID: 28280249 PMC5595217

[34] Patsoukis N Duke-Cohan JS Chaudhri A Aksoylar H-I Wang Q Council A . Interaction of SHP-2 SH2 domains with PD-1 ITSM induces PD-1 dimerization and SHP-2 activation. Commun Biol. (2020) 3:128. doi: 10.1038/s42003-020-0845-0. PMID: 32184441 PMC7078208

[35] Chan W Cao YM Zhao X Schrom EC Jia D Song J . TCR ligand potency differentially impacts PD-1 inhibitory effects on diverse signaling pathways. J Exp Med. (2023) 220:e20231242. doi: 10.1084/jem.20231242. PMID: 37796477 PMC10555889

[36] Lee J-S Sugawara S Kang J-H Kim HR Inui N Hida T . Four‐year outcomes for nivolumab with chemotherapy and bevacizumab in patients with nonsquamous NSCLC in the TASUKI‐52. Cancer Sci. (2026) 117(4):1124–35. doi: 10.1111/cas.70330. PMID: 41611341 PMC13045390

